# Positive Effect of Cold Atmospheric Nitrogen Plasma on the Behavior of Mesenchymal Stem Cells Cultured on a Bone Scaffold Containing Iron Oxide-Loaded Silica Nanoparticles Catalyst

**DOI:** 10.3390/ijms21134738

**Published:** 2020-07-03

**Authors:** Agata Przekora, Maïté Audemar, Joanna Pawlat, Cristina Canal, Jean-Sébastien Thomann, Cédric Labay, Michal Wojcik, Michal Kwiatkowski, Piotr Terebun, Grazyna Ginalska, Sophie Hermans, David Duday

**Affiliations:** 1 Chair and Department of Biochemistry and Biotechnology, Medical University of Lublin, Chodzki 1 Street, 20-093 Lublin, Poland; michal.wojcik@umlub.pl (M.W.); g.ginalska@umlub.pl (G.G.); 2 IMCN Institute, Université catholique de Louvain, Place Louis Pasteur 1, 1348 Louvain-la-Neuve, Belgium; maite.audemar@uclouvain.be; 3 Chair of Electrical Engineering and Electrotechnologies, Lublin University of Technology, Nadbystrzycka 38a, 20-618 Lublin, Poland; askmik@hotmail.com (J.P.); m.kwiatkowski@pollub.pl (M.K.); p.terebun@pollub.pl (P.T.); 4 Biomaterials, Biomechanics and Tissue Engineering Group, Department of Materials Science and Engineering, Universitat Politècnica de Catalunya (UPC), Av. Eduard Maristany 14, 08930 Barcelona, Spain; cristina.canal@upc.edu (C.C.); cedric.labay@upc.edu (C.L.); 5 Barcelona Research Center in Multiscale Science and Engineering, UPC, 08019 Barcelona, Spain; 6 Research Centre for Biomedical Engineering (CREB), UPC, 08019 Barcelona, Spain; 7 Material Research and Technology (MRT) Department, Luxembourg Institute of Science and Technology (LIST), 41 rue du Brill, L-4422 Belvaux, Luxembourg; jean-sebastien.thomann@list.lu

**Keywords:** reactive oxygen species, cold atmospheric plasma, mesoporous silica nanoparticles, biomaterials, bone regeneration, cytotoxicity, proliferation, osteogenic differentiation

## Abstract

Low-temperature atmospheric pressure plasma was demonstrated to have an ability to generate different reactive oxygen and nitrogen species (RONS), showing wide biological actions. Within this study, mesoporous silica nanoparticles (NPs) and Fe_x_O_y_/NPs catalysts were produced and embedded in the polysaccharide matrix of chitosan/curdlan/hydroxyapatite biomaterial. Then, basic physicochemical and structural characterization of the NPs and biomaterials was performed. The primary aim of this work was to evaluate the impact of the combined action of cold nitrogen plasma and the materials produced on proliferation and osteogenic differentiation of human adipose tissue-derived mesenchymal stem cells (ADSCs), which were seeded onto the bone scaffolds containing NPs or Fe_x_O_y_/NPs catalysts. Incorporation of catalysts into the structure of the biomaterial was expected to enhance the formation of plasma-induced RONS, thereby improving stem cell behavior. The results obtained clearly demonstrated that short-time (16s) exposure of ADSCs to nitrogen plasma accelerated proliferation of cells grown on the biomaterial containing Fe_x_O_y_/NPs catalysts and increased osteocalcin production by the cells cultured on the scaffold containing pure NPs. Plasma activation of Fe_x_O_y_/NPs-loaded biomaterial resulted in the formation of appropriate amounts of oxygen-based reactive species that had positive impact on stem cell proliferation and at the same time did not negatively affect their osteogenic differentiation. Therefore, plasma-activated Fe_x_O_y_/NPs-loaded biomaterial is characterized by improved biocompatibility and has great clinical potential to be used in regenerative medicine applications to improve bone healing process.

## 1. Introduction

Plasma is an ionized gas that comprises various molecules, electrons, ions, excited species, and radicals. In the field of engineering of biomaterials and regenerative medicine, special attention has been paid to the application of non-thermal atmospheric pressure plasma, also known as cold plasma, for improving biological properties of the biomaterials and acceleration of healing processes [1,2,3,4,5,6,7,8,9]. In the field of biomaterials engineering, cold plasma technology is primarily used for surface functionalization with hydrophilic chemical groups to enhance cell adhesion and proliferation on the implants [10]. Atmospheric pressure plasma is also used to improve surface properties of the biomaterials, especially wettability and roughness that are known to significantly influence biocompatibility of the implants [11,12]. Materials scientists frequently use atmospheric pressure plasma combined with oxygen, argon, air, ammonia, or nitrogen gas for biocompatibility improvement of various polymeric biomaterials [5,6,7,8]. Atmospheric plasma processes are also used to graft molecules on the surface of biomaterials [13].

Since reactive species formed upon plasma treatment may have a positive effect on cell proliferation and differentiation, cold plasma has also attracted great attention in the regenerative medicine field. Possible applications of atmospheric pressure plasma have been reported for acceleration of wound healing [1,2,3,4] and bone regeneration [14,15]. The effectiveness of plasma technology application in the regenerative medicine field highly depends on the reactor design, the gas nature, the plasma power, and other characteristics of the treatment [11]. It was demonstrated that cold oxygen or nitrogen plasma may generate different reactive oxygen and nitrogen species (RONS) that may significantly affect cell metabolism, proliferation, and differentiation [16]. Importantly, the biological activity of RONS is mediated by the liquid environment surrounding the cells, indicating that reactive species, either short-lived e.g., singlet oxygen (^1^O_2_), hydroxyl (OH) or nitric oxide (NO) radicals, or long-lived i.e., H_2_O_2_ or NO_2_, formed upon plasma treatment in a liquid environment (e.g., phosphate buffered saline, culture medium), have equivalent or superior activities over these generated in the gas phase [17]. Furthermore, comparative studies on the formation of reactive species (^1^O_2_, H radicals, OH radicals, and NO radicals) upon treatment of the phosphate buffered saline (PBS) with oxygen, air, helium, argon, nitrogen, and carbon dioxide plasmas, proved that nitrogen plasma has the ability to produce greater amounts of NO radicals and the largest amount of OH radicals among all tested gas species [17]. Therefore, within our studies, it was decided to use nitrogen as a source gas during plasma treatment of cell-seeded biomaterials.

It is also known that OH radicals may be formed in the liquid phase by the heterogeneous Fenton-like reaction [18] using metal or metal oxides (e.g., Au, Mn, Cu, Co, or Fe oxide) [19,20]. Therefore, herein, catalysts based on two types of silica nanoparticles (NPs) decorated with iron oxide (noted Fe_x_O_y_ to denote magnetite + maghemite phase) as active phase were synthesized to enhance cold plasma-induced generation of RONS. The NPs produced with and without Fe_x_O_y_ decoration were immobilized by the entrapment method within the polysaccharide matrix of a bone scaffold made of hybrid chitosan/curdlan matrix and hydroxyapatite (HA) granules. Then, modified biomaterials were seeded with human adipose tissue-derived mesenchymal stem cells (ADSCs) and subjected to cold atmospheric nitrogen plasma treatment to determine its effect on cell proliferation and osteogenic differentiation. The effect of embedded NPs on the production of OH radicals upon plasma treatment as well as basic mechanical and microstructural properties of the modified biomaterials were also evaluated. The primary goal of the presented study was to assess biomedical potential in regenerative medicine applications of Fe_x_O_y_/NPs-loaded biomaterial pre-seeded with human stem cells and activated by cold atmospheric nitrogen plasma.

## 2. Results and Discussion

### 2.1. Characterization of Produced NPs

Within this study, two types of silica NPs were produced to enhance reactive oxygen species (ROS) generation upon plasma treatment of NPs-loaded bone scaffolds: (1) Mobil Composition of Matter No. 48 (MCM-48) and (2) wormhole mesoporous silica nanoparticles (MSNPs). Produced NPs were subjected to basic characterization of their physicochemical and microstructural properties. The MCM-48 support exhibited a high specific surface area (SSA) of 1440 ± 46 m^2^/g. MSNPs were characterized by lower SSA equal to 930 ± 10 m^2^/g. Obtained nitrogen adsorption/desorption isotherms indicated a mesoporous structure of both NPs with a type IV isotherm. MSNPs were characterized by pore size of 2.8 ± 0.2 nm with a pore volume of 0.61 ± 0.05 cm^3^/g, whereas MCM-48 particles had a pore size distribution centered on an average of 2.7 nm with a pore volume of 0.73 ± 0.05 cm^3^/g (Supplementary Figure S1). These mesoporous silica materials were loaded with iron oxide by dry impregnation with iron nitrate in ethanol followed by reduction at 500 °C under H_2_:Ar (1:4 *V*:*V*) after a calcination step. The reduction temperature was chosen to obtain a mix of Fe(II) and Fe(III), which is known to be the active phase in Fenton chemistry. The iron loading of NPs was measured by inductively coupled plasma (ICP), which gave a value of 4.7 wt.% and 4.9 wt.% for the MCM-48 and MSNPs materials, respectively.

**Figure 1:**
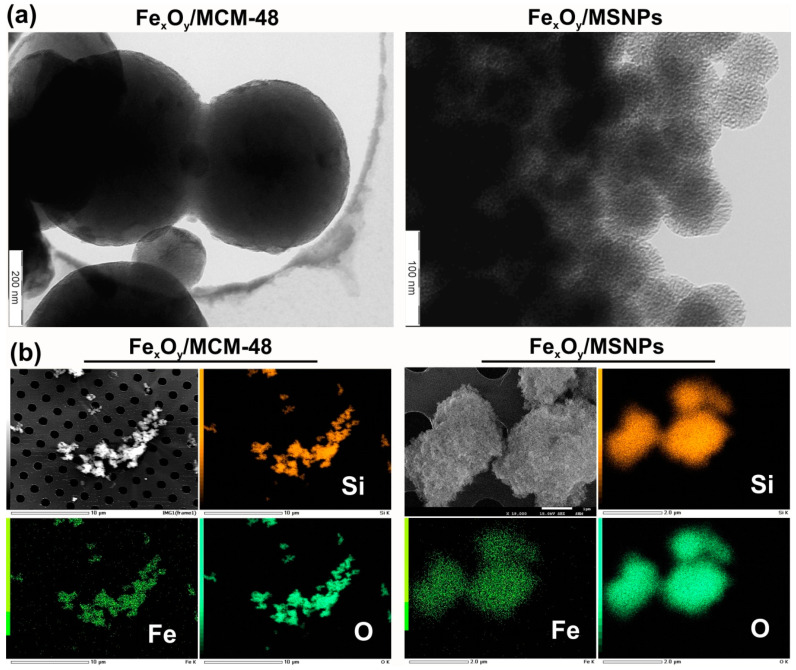
Visualization of produced NPs: (**a**) TEM images of Fe_x_O_y_/MCM-48 and Fe_x_O_y_/MSNPs catalysts obtained by dry impregnation; (**b**) SEM-EDX mapping of Si, Fe, O elements in the Fe_x_O_y_/MCM-48 and Fe_x_O_y_/MSNPs samples.

**Figure 2:**
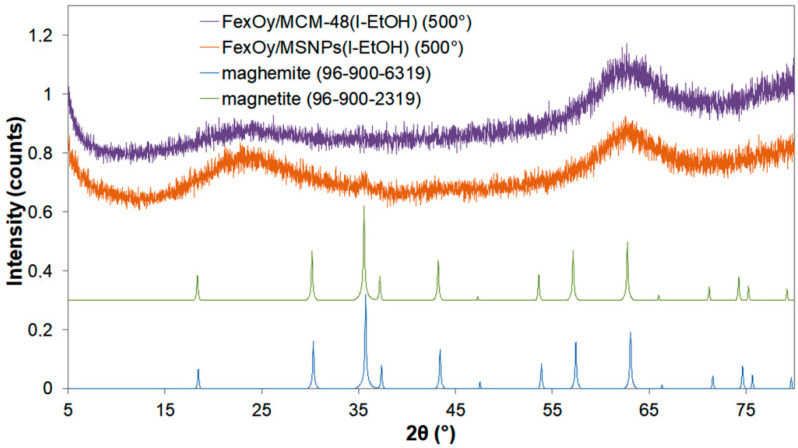
XRD diffractograms of the Fe_x_O_y_/MCM-48 and the Fe_x_O_y_/MNPs materials obtained by dry impregnation and reduced at 500 °C (bump at 2θ = 63–64° is an artefact due to the sample holder) with reference data for magnetite and maghemite phases.

The MSNPs preparation resulted in non-aggregated NPs with a diameter of 55 ± 5 nm (Supplementary Figure S2). MCM-48 NPs presented significantly higher size than MSNPs (around 400 nm). Zeta potential for MSNPs was −30 ± 5 mV, allowing a repulsion between the MSNPs in water. With the dry impregnation method, the iron oxide introduced within the pores of these materials was unsurprisingly not visible on the TEM images (Figure 1a) neither with the MCM-48 nor the MSNPs. Although TEM images of these materials showed no visible iron oxide particles decorating the silica nanoparticles, SEM-EDX mapping of Si, Fe, O showed a homogeneous dispersion of iron within the two different silica nanoparticles, confirming very small iron oxide particles sizes distributed throughout the entire silica (Figure 1b). X-ray photoelectron spectroscopy (XPS) analysis also revealed the presence of iron at the surface of the decorated silica samples (Table 1). However, no signal of iron oxide phases was visible by X-ray diffraction (XRD) for the Fe_x_O_y_/MCM-48 catalyst, due to very small or amorphous iron oxide particles (Figure 2). For the Fe_x_O_y_/MSNPs sample, a small peak at 35.5° was visible on the diffractogram, matching with the most intense diffraction peak of magnetite Fe_3_O_4_ or maghemite γ-Fe_2_O_3_. This suggests that the iron oxide active sites in the case of Fe_x_O_y_/MSNPs were at least partially crystalline and/or slightly bigger particles than within the MCM-48 support.

### 2.2. Biocompatibility Screening Tests on the NPs-Loaded Biomaterials

The produced NPs were incorporated into the polysaccharide matrix of chitosan/curdlan/HA scaffold to improve biocompatibility of the resultant biomaterial by enhancement of ROS formation upon exposure to cold nitrogen plasma. Before performing experiments making use of human ADSCs, aiming at determining the effect of plasma-induced ROS generation on proliferation and osteogenic differentiation of stem cells, biocompatibility screening tests were carried out using mouse calvarial preosteoblasts (MC3T3-E1 Subclone 4) to select a NPs–biomaterial combination that was non-toxic and the most supportive to osteoblast growth. For this purpose, biomaterials comprising three different concentrations (0.25, 0.125, and 0.05 wt.%) of MCM-48 and MSNPs with and without Fe_x_O_y_ were tested.

**Figure 3:**
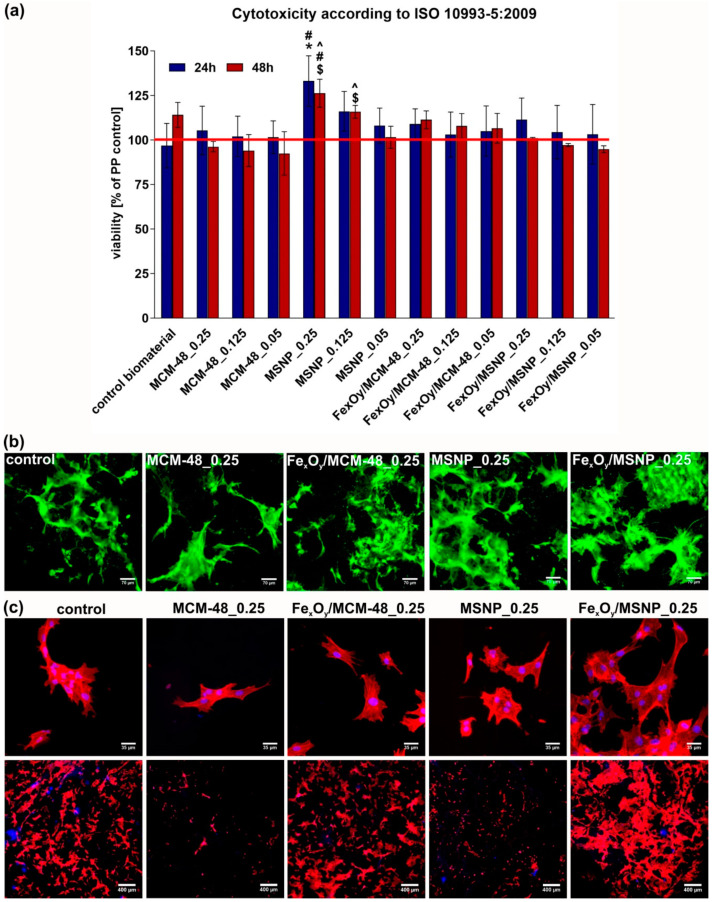
Biocompatibility screening tests on the NPs-loaded biomaterials: (**a**) MTT cytotoxicity test with the use of extracts of the biomaterials (PP control—cells exposed to the extract of polypropylene, revealing 100% viability; control biomaterial—extract of the biomaterial without any NPs; 0.25, 0.125, 0.05—concentration (wt.%) of NPs within the structure of the biomaterial; * statistically significant differences compared to the control biomaterial, # compared to the biomaterial containing Fe_x_O_y_/MCM-48 at corresponding concentration; $ compared to the biomaterial containing MCM at a corresponding concentration, and ^ compared to the biomaterial containing Fe_x_O_y_/MSNPs at a corresponding concentration, according to One-way Anova followed by Tukey’s test, *p* < 0.05); (**b**) CLSM images of 3-day culture of preosteoblasts (MC3T3-E1 cells at high concentration were seeded) on the surface of the biomaterials upon fluorescent live/dead staining (control—biomaterial without any NPs; 0.25—concentration (wt.%) of NPs within the structure of the biomaterial; green fluorescence—viable cells, red fluorescence—nuclei of dead cells, magn. 200×); (**c**) CLSM images of 3-day culture of preosteoblasts (MC3T3-E1 cells at low concentration were seeded) on the surface of the biomaterials upon fluorescent staining of cytoskeleton and nuclei (red fluorescence—cytoskeleton, blue fluorescence—nuclei, upper images—magn. 400×, lower images—magn. 40×).

MTT cytotoxicity test performed according to ISO 10993-5 (with the use of the extracts of the biomaterials) revealed that all NPs-loaded biomaterials were non-toxic to MC3T3-E1 preosteoblasts throughout the full length of the experiment (Figure 3a). Interestingly, biomaterials comprising MSNPs (without the Fe_x_O_y_ active phase) at the concentrations of 0.25 wt.% and 0.125 wt.% significantly increased cell metabolism (thus viability) compared to other samples. Based on MTT test results, biomaterials containing NPs at the highest concentration (0.25 wt.%) were selected for further experiments with the use of confocal laser scanning microscope (CLSM). Live/dead staining and observation by confocal microscopy of the MC3T3-E1 preosteoblasts seeded at high concentration onto the surface of the samples confirmed non-toxicity of all tested biomaterials (Figure 3b). Preosteoblasts were well spread on the surface of the scaffolds and emitted only green fluorescence, indicating their high viability. Nevertheless, there were noticeably fewer cells on the biomaterial containing MCM-48 particles.

To select the variant of the biomaterial that is the most supportive to osteoblast growth and proliferation, preosteoblasts were seeded onto the biomaterials at low concentration and cultured for 3 days. Then, CLSM observation upon fluorescent staining of cytoskeleton was performed. CLSM images clearly demonstrated that biomaterials comprising both tested NPs without Fe_x_O_y_ decoration negatively affected cell growth since there were meaningfully fewer cells on their surfaces compared to the control biomaterial without NPs (Figure 3c). Nevertheless, although biomaterials containing NPs without Fe_x_O_y_ inhibited cell growth, higher magnification images showed that surfaces of all scaffolds allowed for good cell adhesion and spreading. Interestingly, addition of Fe_x_O_y_ to the NPs overcame the negative effect of NPs without iron oxide decoration. The surface of the biomaterial with incorporated Fe_x_O_y_/MCM-48 was characterized by similar cell number to control biomaterial (without any NPs), whereas biomaterial containing Fe_x_O_y_/MSNPs noticeably improved cell growth and spreading. Based on the results obtained with the screening biocompatibility tests, bone scaffolds comprising Fe_x_O_y_/MSNPs and pure MSNPs (as a reference sample) at the concentration of 0.25 wt.% were selected for further experiments.

### 2.3. Characterization of the Selected NPs-Loaded Biomaterials

Based on the screening biocompatibility tests, MSNPs-loaded biomaterials were selected as the most promising. However, bone scaffolds for regenerative medicine applications should possess some key microstructural features, such as high stability, good compressive strength, or high porosity (at least 40%), allowing for new blood vessel formation and bone ingrowth deep into the implant [21,22,23,24]. Therefore, basic microstructural characterization of the scaffolds was performed to check whether incorporation of MSNPs and Fe_x_O_y_/MSNPs catalyst into the biomaterial structure affected its mechanical properties and porosity. Moreover, distribution of MSNPs within the polysaccharide matrix of the scaffolds was visualized by SEM.

**Figure 4:**
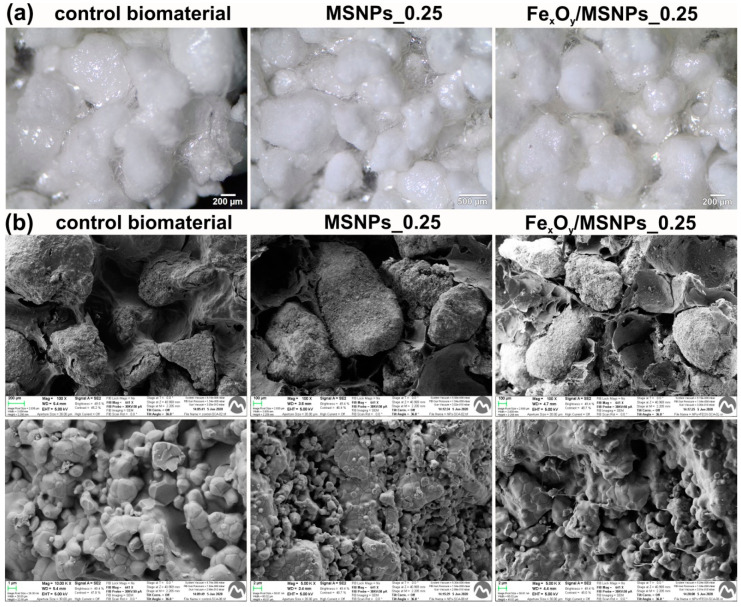
Visualization of the produced MSNPs-loaded scaffolds: (**a**) stereoscopic microscope images of the materials; (**b**) SEM micrographs of the scaffolds (upper images—magn. 100×, lower images—magn. 10000× for control and 5000× for NPs-loaded biomaterials).

The control scaffold (without any MSNPs) was composed of biopolymers and 80 wt.% HA granules, and on the macroscopic scale displayed the same morphology as biomaterials with the addition of MSNPs or MSNPs decorated with Fe_x_O_y_ (Figure 4a). SEM observation made it possible to characterize the HA granules according to the structure of typical sintered ceramics, which were bound by a continuous polymer phase (Figure 4b) with a high degree of porosity that was estimated to be approximately 50% in all samples, as measured by Mercury Intrusion Porosimetry (MIP) (Table 2).

**Figure 5:**
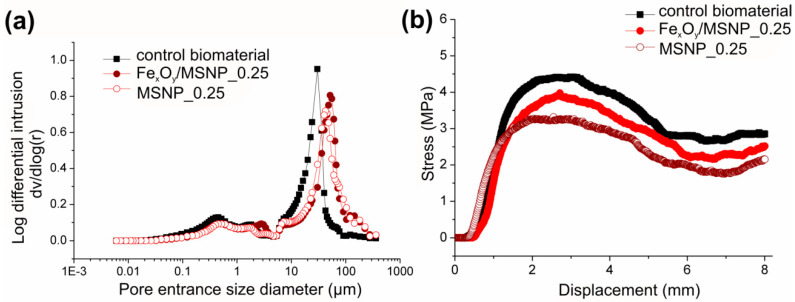
Basic characteristics of NPs-loaded biomaterial: (**a**) pore size distribution of the three different materials as determined by MIP; (**b**) stress–strain curves obtained with compression testing for the control biomaterial and scaffolds loaded with NPs and Fe_x_O_y_/NPs.

As expected, the total porosity of the material remained unaltered with the incorporation of NPs and the Fe_x_O_y_ decoration at a concentration of 0.25 wt.% (Table 2), while the pore size distribution was slightly affected, displaying a shift of the main peak to larger pore sizes (Figure 5a). Thus, the Fe_x_O_y_/MSNPs_0.25 presented a slightly lower volume of pores but at larger entrance sizes compared to the control biomaterial.

Compression testing revealed that the three materials displayed strain hardening curve, characteristic of ductile composites, in agreement with the SEM images (Figure 5) showing a continuous polymeric phase with embedded HA. The fracture toughness or work of fracture (WOF), obtained from compression tests presented in Figure 5b, tended to decrease with the incorporation of the NPs and Fe_x_O_y_; however, no statistical differences can be pointed out between the different samples. The elastic modulus of the materials was also slightly decreasing with the incorporation of the different NPs; however, despite the known importance of biomechanics, no change should be expected in cell behavior from this low variation (Table 2).

### 2.4. Plasma-Induced Production of OH radicals by NPs-Loaded Biomaterials

The main concept of this study was to incorporate Fe_x_O_y_-based catalysts into the structure of the biomaterials to enhance short-lived ROS and RNS formation upon nitrogen plasma treatment that would subsequently positively affect stem cell behavior. During our pilot studies, the effect of air and nitrogen plasma on viability of mouse preosteoblasts (MC3T3-E1 cell line) was determined. It was observed that longer exposure time (>16 s) had a negative effect on cell viability. Furthermore, cells revealed higher viability after 16 s exposure to nitrogen plasma compared to the air plasma. In our another study [15], it was demonstrated that 16 s nitrogen plasma treatment promoted preosteoblasts proliferation and enhanced their osteogenic differentiation. Importantly, the best results were obtained when MC3T3-E1 preosteoblasts were left in Hanks’ Balanced Salt solution (HBSS) for 3 h after plasma exposure. Thus, on the basis of our previous experiments, in this study it was decided to use nitrogen as a source gas for plasma treatment and 16 s exposure time, followed by 3 h maintenance of the cells in HBSS after plasma treatment.

**Figure 6:**
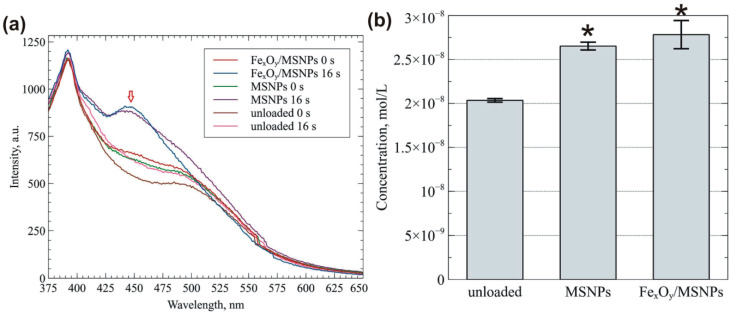
Plasma-induced OH production by biomaterials: (**a**) emission intensities of tested scaffolds (an arrow indicates emission peak of umbelliferone); (**b**) umbelliferone concentrations in HBSS solutions after 16 s GAD plasma treatment (* significantly different results compared to the control biomaterial (unloaded), according to One-way Anova followed by Tukey’s test, *p* < 0.05).

Based on the biocompatibility tests, Fe_x_O_y_/MSNPs catalyst was selected as the most promising material for bone therapies. Presence of Fe_x_O_y_ in biomaterial suspended in water solution and treated by non-thermal plasma should enhance formation of oxygen-based active species, especially OH radicals in accordance to Fenton reaction mechanism [25]. OH radical production upon GlidArc (GAD) plasma treatment of the control scaffold (without nanoparticle addition) and scaffolds loaded with MSNPs and Fe_x_O_y_/MSNPs was determined by spectrofluorimetric measurements of the highly fluorescent probe umbelliferone, which is the product of coumarin and OH radical reaction [26]. Scaffolds suspended in HBSS were treated with plasma for 16 s in an analogous manner as cell-seeded biomaterials in the subsequent tests. Gliding arc is a versatile source, which allows working with pure nitrogen gas and renders relatively large area in comparison to helium/argon jet sources. For such a short treatment time, the control unit for the power supply with the timer was developed. First, gas flow was set using gas flow regulator, then the electrical discharge was ignited via control unit and it lasted for 16 s, then the power supply was shut down. The emission intensities of tested samples are presented in Figure 6a. The characteristic peak at 452 nm related to the presence of umbelliferone after short plasma treatment was clearly observed for the HBSS solutions with samples loaded with NPs. In the case of control scaffold (without any NPs), extremely low concentrations of generated active species were probably instantly consumed on-site by the surface reactions with organic part of biomaterial itself and they were almost below the detection limit in the liquid. Figure 6b depicts concentrations of umbelliferone in HBSS solution after 16 s of plasma treatment. The amount of generated OH radicals was low, but as expected, the umbelliferone concentrations obtained for Fe_x_O_y_/MSNPs-loaded biomaterial were slightly higher than for the MSNPs-loaded sample and control material.

It should be noted that NPs were incorporated by the entrapment method within the hydrogel matrix of the biomaterial. It is very likely that most OH radicals formed upon plasma exposure were entrapped within the hydrogel matrix and their release to the HBSS was hindered. Therefore, very low concentrations of OH radicals were detected in HBSS liquid medium. Nevertheless, it is assumed that high concentrations of reactive species could be present within the hydrogel matrix of the biomaterial directly affecting behavior of cells cultured on its surface. Because the plasma was not touching the HBSS and N_2_ plasma gas was used for the treatment, it may also be assumed that a large quantity of NO_2_/NO could be formed in our conditions [27]. As OH radicals are generated on Fe_x_O_y_ nanocatalyst more efficiently than NO, both types of radicals are expected to be present at similar and high quantities in our conditions.

### 2.5. Plasma Effect on Stem Cell Behavior Cultured on the NPs-Loaded Biomaterials

Plasma treatment of the MSNPs- and Fe_x_O_y_/MSNPs-loaded biomaterials seeded with the human ADSCs was performed in Hanks’ Balanced Salt solution (HBSS) to avoid unexpected results due to the high complexity of the culture medium [28]. Stem cells-seeded biomaterials were exposed to cold nitrogen plasma for 16 s and left for 3 h in HBSS after plasma treatment. These conditions were demonstrated in our previous studies to be very effective in stimulation of proliferation and ECM synthesis by MC3T3-E1 preosteoblasts [15].

**Figure 7:**
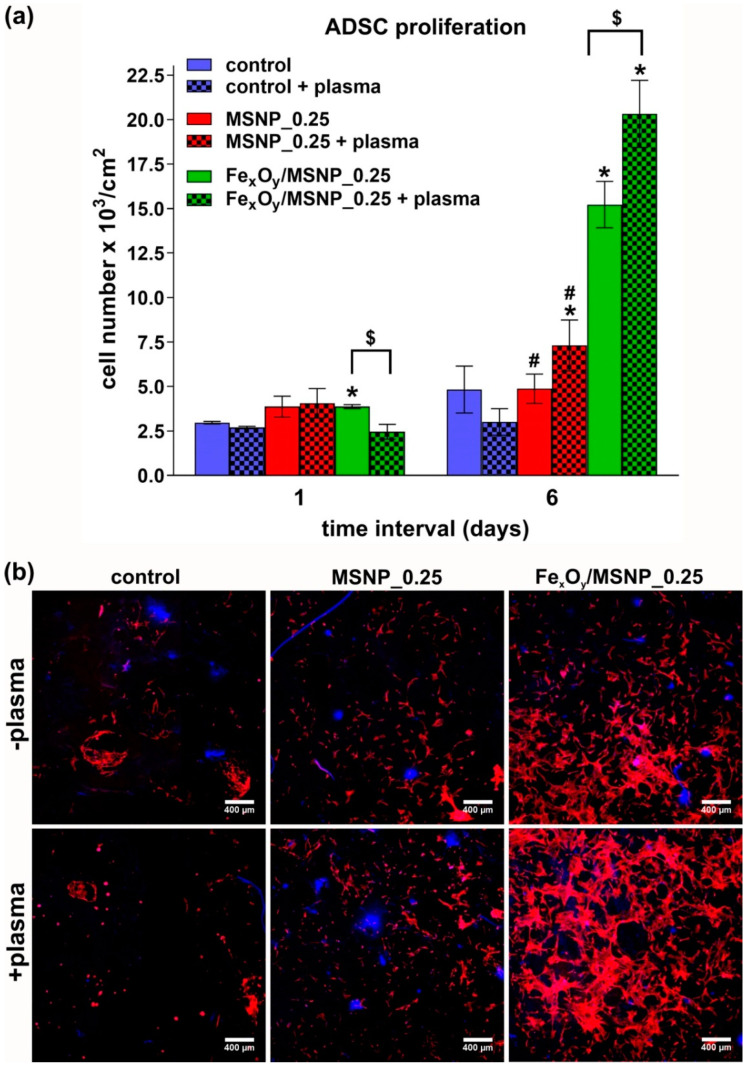
Proliferation of ADSCs on the surface of the NPs-loaded scaffolds upon nitrogen plasma exposure (16 s in HBSS; cells left in HBSS for 3 h after plasma treatment): (**a**) cell number increase with time determined by nuclei counting (control—biomaterial without any NPs; 0.25—concentration (wt.%) of NPs within the structure of the biomaterial; * statistically significant results compared to the corresponding (with or without plasma activation) control biomaterial, # compared to the corresponding (with or without plasma activation) biomaterial containing Fe_x_O_y_/MSNPs; $ statistically significant results between plasma-treated and untreated biomaterials, according to One-way Anova followed by Tukey’s test, *p* < 0.05); (**b**) CLSM images of ADSCs on the surface of the biomaterials (cells at extremely low concentration were seeded) 6 days after plasma treatment upon fluorescent staining of cytoskeleton and nuclei (red fluorescence—cytoskeleton, blue fluorescence—nuclei, magn. 40×).

To determine the effect of nitrogen plasma on ADSC proliferation on the biomaterials, stem cells were seeded directly on the samples at extremely low concentration. Within this study, it was clearly demonstrated that ADSCs proliferated significantly faster on the surface of plasma-activated biomaterials with incorporated MSNPs and Fe_x_O_y_/MSNPs catalyst compared to the plasma-activated control biomaterial (without any NPs) (Figure 7a).

Importantly, 6 days after plasma exposure, there were 3-fold more cells on the biomaterials with Fe_x_O_y_/MSNPs compared to the MSNPs-loaded scaffolds regardless of the plasma activation, indicating that the presence of the active phase (Fe_x_O_y_) itself was a key factor responsible for the enhancement of cell proliferation. This indicates that Fe_x_O_y_/MSNPs catalyst incorporated into the structure of the biomaterial had by itself the ability (without plasma activation) to induce radical formation by the heterogeneous Fenton-like reaction at sufficient level to improve cell proliferation. CLSM images of ADSCs grown on the biomaterials confirmed meaningfully greater number of cells on the biomaterials comprising Fe_x_O_y_ active phase compared to other samples (Figure 7b). Plasma exposure of ADSCs grown on the Fe_x_O_y_/MSNPs-loaded biomaterial resulted in significant increase in cell number compared to the untreated Fe_x_O_y_/MSNPs-loaded sample (doubling time = 38.96 h for plasma-activated cells and doubling time = 60.74 h for untreated cells) (Figure 7a). Therefore, oxygen-based active species, more particularly OH radicals, generated upon plasma activation of Fe_x_O_y_/MSNPs-loaded biomaterial reached higher concentration compared to untreated sample, thereby significantly accelerating proliferation of mesenchymal stem cells. This is in agreement with previous works where other multipotent cells (i.e., human bone marrow-derived mesenchymal stem cells) displayed enhanced proliferation under controlled amounts of ROS generated by plasmas [29], and the differences observed after 6 days (Figure 7a) are clearly in agreement with the observed “delayed” effect of cold plasmas, where an initial dose of ROS is able to trigger long term effects on cells [28,29].

**Figure 8:**
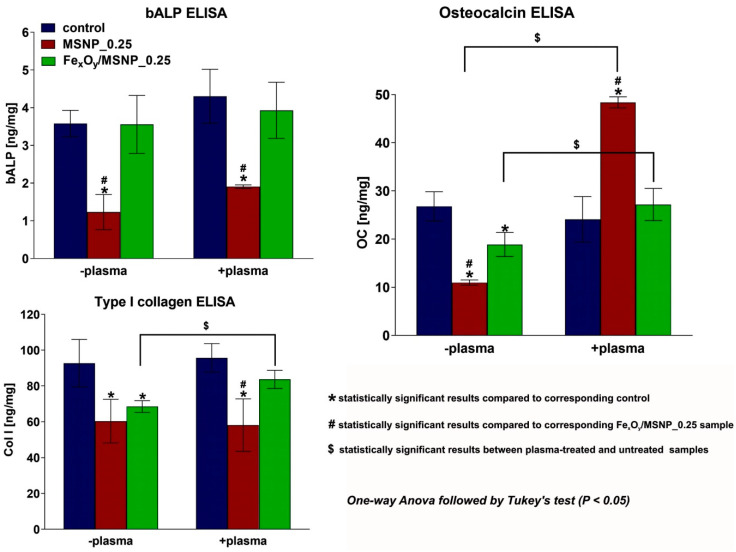
Osteogenic differentiation of ADSCs on the surface of the NPs-loaded scaffolds determined 20 days after nitrogen plasma exposure (16 s in HBSS; cells left in HBSS for 3 h after plasma treatment); control—biomaterial without any NPs; 0.25—concentration (wt.%) of NPs within the structure of the biomaterial.

Osteogenic differentiation of ADSCs cultured on the plasma-activated scaffolds was determined by detection of typical markers of bone formation process: bone alkaline phosphatase (bALP), type I collagen (Col I), and osteocalcin (OC). The experiment revealed that incorporation of MSNPs into the biomaterial structure significantly inhibited bALP production by ADSCs regardless of plasma activation (Figure 8). However, addition of Fe_x_O_y_ overcame this negative effect of pure MSNPs, thus stem cells grown on the Fe_x_O_y_/MSNPs-loaded scaffold showed similar bALP level to control samples. Col I synthesis by ADSCs was significantly reduced in the presence of both MSNPs and Fe_x_O_y_/MSNPs compared to the control biomaterial. However, this effect was slightly overcome by plasma activation of the Fe_x_O_y_/MSNPs-loaded scaffold, whereas plasma treatment of MSNPs-loaded biomaterial did not influence Col I production. Similarly, OC production was significantly decreased by both MSNPs- and Fe_x_O_y_/MSNPs-loaded biomaterials. In this case, plasma activation significantly increased OC synthesis by ADSCs on both mentioned biomaterials compared to untreated samples. Surprisingly, plasma treatment of Fe_x_O_y_/MSNPs-loaded biomaterial only overcame negative effect of the Fe_x_O_y_/MSNPs catalyst on the OC production, whereas plasma activation of MSNPs-loaded scaffold resulted in 5-fold increase in OC level compared to untreated MSNPs-loaded biomaterial and 2-fold increase compared to the control biomaterial (plasma-treated and untreated). Importantly, no statistically significant differences between plasma-treated and untreated control samples—with respect to bALP, Col I, and OC production—were observed.

In our previous studies performed on MC3T3-E1 preosteoblasts, it was demonstrated that short-time (16 s) nitrogen plasma treatment of the cells significantly promoted cell proliferation and enhanced bALP and OC synthesis [15]. In this study, nitrogen plasma activation of control biomaterial (without NPs) seeded with ADSCs did not influence neither cell proliferation nor osteogenic differentiation. It indicates that ADSCs are less sensitive to plasma long-lived reactive species (e.g., H_2_O_2_) than preosteoblast cells. Nevertheless, plasma activation of Fe_x_O_y_/MSNPs-loaded biomaterial resulted in significantly accelerated cell proliferation (Figure 7). Thus, the proliferation of ADSCs seems to be more sensitive to short-lived reactive species (like OH radicals) provided by the presence of Fe_x_O_y_ nanocatalysts on the MSNPs. Enhanced proliferation of ADSCs on the scaffold comprising Fe_x_O_y_/MSNPs upon exposure to nitrogen plasma may be explained by short-lived reactive species-mediated induction of fibroblast growth factor-2 (FGF-2) release by the cells. This mechanism of plasma-induced accelerated cell proliferation was proposed by Kalghatgi et al., who suggested that O_3_, NO, H_2_O_2_, or OH generated upon plasma activation may lead to increased FGF-2 release [30]. Here, it was shown that short-lived reactive species and more probably OH radicals were produced in higher quantities, due to the combination of plasma activation and the presence of Fenton supported nanocatalysts, and were the key players for this mechanism. In turn, increased levels of FGF-2 in the microenvironment was an autocrine or paracrine signal for mesenchymal stem cells, influencing their proliferation [31,32,33]. Therefore, it indicates that accelerated proliferation of ADSCs observed in our studies resulted from enhanced production of active species, inducing local formation of OH radicals on the Fe_x_O_y_ catalysts upon plasma activation of Fe_x_O_y_/MSNPs-loaded biomaterial.

It is known that Si plays an important role in several bone biochemical processes occurring in the living organism, including promotion of osteogenic differentiation [34]. However, incorporation of MSNPs with and without Fe_x_O_y_ decoration had a negative effect on osteogenic differentiation of ADSCs. This effect could be the result of the release of a certain amount of MSNPs and Fe_x_O_y_/MSNPs when the scaffolds were swelling in the aqueous environment (typical behavior of hydrogel type biomaterials) [23]. The attachment of free NPs on the cell membrane could alter the cell differentiation activity. To solve this issue, MSNPs could be covalently attached (instead of immobilization by entrapment) to the glucan matrix or hydroxyapatite granules to better control their release and avoid a burst release effect.

ADSCs cultured on the Fe_x_O_y_/MSNPs-loaded biomaterial showed slightly better osteogenic potential than cells on MSNPs-biomaterial (except OC synthesis upon plasma exposure) (Figure 8). Thus, the generation of short-lived species, mainly OH radicals, from the intrinsic H_2_O_2_ and at a lower extent the possible slow release of Fe ions from the Fe_x_O_y_/MSNPs-loaded biomaterial were shown to overcome negative effect of MSNPs on osteogenic differentiation. It is known that Fe is crucial for many biochemical reactions and processes occurring in the living organism, including oxygen transport and enzymatic reactions. Interestingly, recent studies have also demonstrated close correlation between iron supply and bone metabolism [35,36,37].

Without plasma treatment, ADSCs cultured on the NPs-loaded biomaterials generally revealed reduced osteogenic activity compared to the control biomaterial. However, upon plasma treatment of cells grown on the Fe_x_O_y_/MSNPs-loaded biomaterial, osteogenic markers (bALP, Col I, and OC) were at the same level as in the control sample. In the case of plasma-treated ADSCs cultured on the MSNPs-loaded biomaterial, bALP and Col I markers were reduced, whereas OC increased compared to the control biomaterial (Figure 8). Importantly, Tominami et al. demonstrated that activation of MC3T3-E1 preosteoblats with cold atmospheric helium plasma, which generates mainly H_2_O_2_, OH, and superoxide anion radicals, led to significantly increased bALP level and enhanced mineralization activity of the cells [14]. This indicates that the quantity of reactive species needs to be higher to induce an increase of bALP and Col I markers. However, surprisingly, a large increase of OC production by ADSCs was observed when plasma activation was combined with MSNPs free of Fe_x_O_y_. It seems that the quantity of long-lived reactive species generated in our conditions were close to the optimal one for OC production. Arai et al. suggested that H_2_O_2_ may up-regulate antioxidant system affecting expression of osteogenic genes, including enhancement of OC production [16]. Thus, a lower amount of reactive species seems to be needed to activate the expression of OC. Reduced OC synthesis by ADSCs grown on the Fe_x_O_y_/MSNPs-loaded scaffold compared to catalyst-free biomaterial (in the presence of plasma activation, Figure 8) was even more surprising result. In this case, the quantity of reactive species (short-lived and long-lived) was probably too high and the OC expression was disturbed. Other processes, which were in competition with the OC synthesis, were probably activated due to the high level of oxidative stress reached by combining plasma, MSNPs, and Fe_x_O_y_ catalyst. Thus, a shorter plasma irradiation is needed to stay at the optimum dose of reactive species generated.

## 3. Materials and Methods

### 3.1. Silica NPs Synthesis

Spherical mesoporous Mobil Composition of Matter No. 48 (MCM-48) support was synthesized according to the procedure described by Schumacher et al. [38]. The template n-hexadecyltrimethylammonium bromide (2.4 g) (Sigma-Aldrich Chemicals, Steinheim, Germany) was dissolved under stirring in a mixture of deionized water/ethanol (50 mL/50 mL), followed by the addition of aqueous ammonia (28–30 wt.%, 12 mL) (ChemLab, Zedelgem, Belgium) and this solution was left under stirring for 10 min. Then, the silica source tetraethyl orthosilicate (3.4 g) (Sigma-Aldrich Chemicals, Steinheim, Germany) was added and the mixture was stirred for 2 h under constant stirring. The resulting solid was filtered out, washed with distilled water and dried in air at room temperature. The MCM-48 support was obtained by calcination at 823 K in a muffle furnace under static air, for 6 h, to remove the template.

Wormhole-like mesoporous silica nanoparticles (MSNPs) with interconnected porosity were synthesized using surfactant directed, base-catalyzed condensation of silica precursors with a sol–gel approach proposed earlier by Bein and co-workers [39,40]. The stock solution was prepared by mixing 13.75 mL (762.8 mmol) of milliQ water, 2.23 mL (38.2 mmol) of absolute ethanol, and 2.23 mL (1.69 mmol) of 25% CetylTrimethylAmmonium chloride (CTAC) by stirring in Radleys Mya 4^®^ station for 10 min under Argon atmosphere. Then, TriEthanolAmine (TEA) (1.78 mL; 13.37 mmol) was added and mixed with the stock solution until complete dissolution. When TEA was fully dissolved, the mixture was heated at 60 °C, and then tetraethyl orthosilicate (TEOS) (1.454 mL; 6.5 mmol) was added drop by drop. The reaction was further stirred for 2 h, under Argon atmosphere. The molar ratio of this reaction was: TEOS/CTAC/TEA/H2O/EtOH 1/0.26/2/117.35/5.88. Extraction of the CTACl surfactant was achieved by applying several cycles of hydrochloric acid wash/centrifugation. Each extraction cycle included a dispersion of MSNPs in a solution of ethanol and hydrochloric acid at 2%, and an ultrasonication treatment for 40 min at 40 °C followed by centrifugation for 20 min at 45,000× *g*. After surfactant extraction, MSNPs were either dispersed in absolute ethanol or freeze-dried to obtain a dry powder. All reagents needed for the synthesis of MSNPs were purchased from Sigma-Aldrich Chemicals (Steinheim, Germany).

#### 3.1.1. Preparation of Iron Oxide-Loaded NPs by Dry Impregnation

The NPs were impregnated with an ethanolic solution of Fe(NO_3_)_3_ 9H_2_O (Alfa Aesar, Kandel, Germany), the concentration of which being adjusted to obtain a metal loading of 5 wt.%. Under these conditions, the volume of impregnation solution ensures the complete wetting of the support (by the process known as Incipient Wetness Impregnation, IWI). The impregnated powder was dried at 373 K for 12 h in stagnant air and then calcined at 773 K in a muffle oven. The iron oxide-based catalysts were then reduced at the desired temperature (773 K) under an H_2_ 5%/Ar flow.

#### 3.1.2. Characterization of NPs

The nitrogen adsorption–desorption isotherms were obtained at 77 K with an ASAP-2020 Micrometrics instrument (Micromeritics, Norcross, Georgia, USA), allowing determination of specific surface area (SSA). Before analysis, the samples (0.02–0.10 g) were degassed under 0.133 Pa pressure for 2 h at 200 °C (10 °C/min). The specific surface areas were provided by the analysis of the isotherms with the Brunauer–Emmett–Teller (BET) equation, while the pores’ average diameter was estimated using the DFT model. Zeta potential of nanoparticles were obtained with a Zetasizer Nano (Malvern^®^ Panalytical, Malvern, UK) in deionized water.

Fe_x_O_y_/NPs were visualized by TEM. Images were obtained on a LEO 922 Omega Energy Filter Transmission Electron Microscope (LEO Electron Microscopy Inc., Cambridge, UK, now: Carl Zeiss NTS GmbH) operating at 120 kV. The samples were first suspended in acetone under ultrasonic treatment. A drop of the suspension was deposited on a holey carbon film supported on a copper grid (Holey Carbon Film 300 Mesh Cu, Electron Microscopy Sciences, Hatfield, PA, USA), which was dried overnight under vacuum at room temperature, before introduction in the microscope. The iron loading was measured by ICP on an ICAP 6500 instrument (Thermo Scientific, Cambridge, UK). Before analysis, the samples (known amount) were prepared by acid digestion process.

XRD was performed on a Bruker D8 advanced diffractometer with a Bragg Brentano geometry, using a LinkEye XE-T detector with Cu Kα radiation (λ = 0.15418 nm) and a power of 1200 W (40 kV, 30 mA). The samples were scanned from 0.8 to 10 (2θ range) at a scanning rate of 1.5 °C/min.

X-ray photoelectron spectroscopy (XPS) analyses were carried out at room temperature with a SSI-Xprobe (SSX 100/206) photoelectron spectrometer from Surface Science Instruments (Mountain View, California, USA), equipped with a monochromatized microfocus Al X-ray source. Samples were stuck onto small sample holders with double-face adhesive tape and then placed on an insulating Macor^®^ ceramic carousel. Charge effects were avoided by placing a nickel grid above the samples and using a flood gun set at 8 eV. The binding energies were calculated with respect to the C-(C, H) component of the C1s peak fixed at 284.8 eV. Data treatment was performed using the CasaXPS program (Version 2.3.17dev6.0b, Casa Software Ltd., Teignmouth, UK). The peaks were decomposed into a sum of Gaussian/Lorentzian (85/15) after subtraction of a Shirley-type baseline.

SEM and SEM-EDX (Energy Dispersive X-ray Spectrometry analysis) analyses were performed using a JEOL FEG SEM 7600F (JEOL, Tokyo, Japan) equipped with an EDX system (Jeol JSM2300 with a resolution < 129 eV) operating at 15 keV with a working distance of about 8 mm. The acquisition time for the chemical spectra lasted 300 s with a probe current of 1 nA. The quantitative analysis of the atomic elements was performed with the integrated Analysis Station software. A two-step analysis procedure was applied in order to obtain quantitatively reliable results for the elements profiles over the entire cross sections: (i) the subtraction of the bremsstrahlung done with the classical “Top Hat Filter” method [41,42] and (ii) the quantification of the area under each atomic peak determined by the φ (ρz) model [43,44,45].

### 3.2. Fabrication of NPs-Loaded Biomaterials

The chitosan/curdlan/HA composite was produced according to the procedure described previously by [23,46,47] with some modifications. Krill chitosan (1174 kDa molecular weight, 73% deacetylation degree) was obtained from National Marine Fisheries Research Institute (Gdynia, Poland), whereas curdlan (β-1,3-glucan) was purchased from Wako Pure Chemicals Industries (Osaka, Japan). Suspension of various NPs (with and without Fe_x_O_y_) was prepared in distilled water at the following concentrations: 0.50 wt.%, 0.25 wt.%, and 0.10 wt.%. Then, 16 wt.% curdlan suspension was prepared in the appropriate NPs suspension or in distilled water (control biomaterial) and mixed 1:1 with 4 wt.% chitosan solution prepared in 1% acetic acid solution (Avantor Performance Materials, Gliwice, Poland). The final concentrations of the individual components in the blend were as follow: 8 wt.% curdlan, 2 wt.% chitosan, and 0.25 wt.%, 0.125 wt.% or 0.05 wt.% NPs. The 80 wt.% (*w*/*v*) hydroxyapatite granules (HA BIOCER, Chema Elektromet, Rzeszow, Poland) were added to the blend and the resultant paste was subjected to thermal gelation at 95 °C for 20 min., followed by neutralization in sodium hydroxide (Avantor Performance Materials, Gliwice, Poland).

### 3.3. Characterization of NPs-Loaded Biomaterials

Fabricated biomaterials containing MSNPs or Fe_x_O_y_/MSNPs were visualized by SEM and optical microscope. Pictures of the samples were taken with a stereoscopic microscope (Olympus SZ61TR, Olympus Polska Sp. z o. o., Warsaw, Poland). To study the microstructure of the scaffolds, cross-sections of the samples were made by cutting the cylinder-shaped samples in their middle. A Zeiss Neon 40 cross-beam workstation with Gemini SEM column (Carl Zeiss Iberia, S.L, Madrid, Spain) was used for SEM observation at 5 keV. Prior C-coating of the samples was performed using an EMITECH K950X Turbo Evaporator (Quorum Technologies Ltd., Lewes, UK). SEM images were recorded at magnification range from 100× to 10000× using a secondary electron detector.

Mechanical properties of the biomaterials were assessed by compression testing that was performed using a Bionix^TM^ Test System Model 858 (MTS, Eden Prairie, Minnesota, USA) with an axial load cell of 2.5 kN and a MTS FlexTest Model 40 controller. Compression rate was of 1 mm/min with a maximum piston extension (compression) of 8 mm, considering d = 0 when touching the sample. Monitoring of the system was done by Station Manager software provided by MTS. Three replicates were performed for each kind of samples set in vertical position, and results were presented by plotting the stress (MPa) as a function of the extension (mm).

Mercury Intrusion Porosimetry (MIP, AutoPore IV, Micromeritics, Norcross, Georgia, USA) was carried out to assess the pore entrance size distribution (PESD) within the biomaterials. For each MIP experiment 3-4 cylindrical samples were measured.

### 3.4. Plasma-Induced Production of OH Radicals by NPs-Loaded Biomaterials

For determination of OH radicals’ presence, 40 mg of control scaffold (chitosan/curdlan/hydroxyapatite) and biomaterials loaded with MSNPs or Fe_x_O_y_/MSNPs were immersed in 5 mL of 1 × 10^−3^ mol/L coumarin solution in HBSS. 99% purity coumarin and HBSS were purchased from ACROS Organics™ (Geel, Belgium) and Sigma-Aldrich Chemicals (Warsaw, Poland), respectively. Umbelliferone for calibration was purchased from Sigma-Aldrich (Steinheim, Germany). Spectrofluorimetric measurements of umbelliferone as fluorescent probe were performed with Shimadzu RF-6000 spectrofluorimeter with excitation and emission wavelengths at 346 nm and 452 nm, respectively.

**Figure 9:**
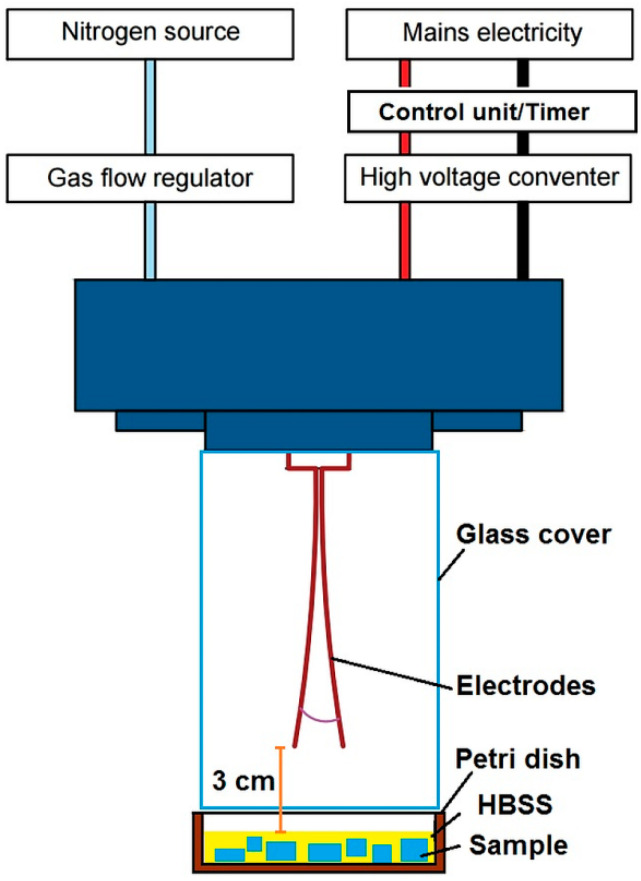
Scheme presenting plasma treatment set-up.

Atmospheric pressure plasma was generated in GAD reactor presented in Figure 9 developed at Lublin University of Technology (LUT), consisting of two copper electrodes (1.5 mm thick, and 10 cm long with 12° angle between them), which has been described in detail in other related publications [15,48]. The smallest inter-electrode distance was 3 mm. The AC high-voltage power supply was operated at 50 Hz frequency. Maximum apparent power was 52 VA and 25 VA on primary and secondary side of transformer, respectively. The RMS voltage and current on the secondary side ranged 688 V and 36 mA, respectively. Current/voltage waveforms may be seen in Supplementary Figure S3. The reactor was supplied with nitrogen as a substrate gas via gas flow regulator at flow rate of 7.33 dm^3^/min. The electrode tips were positioned at 3 cm distance from the surface of the liquid with immersed treated samples. Operation of power supply within selected treatment time of 16 s was controlled using control unit with timer designed at LUT. Measurements were performed in triplicates at room temperature and atmospheric pressure. The same conditions of plasma activation were applied in all cell culture tests.

### 3.5. Cell Culture Experiments

Screening cell culture tests aiming at selecting the most promising NPs-loaded biomaterials were performed using mouse calvarial preosteoblast cell line (MC3T3-E1 Subclone 4, ATCC-LGC standards, Teddington, UK). These were cultured in alpha MEM medium (Gibco, Life Technologies, Carlsbad, California, USA) supplemented with 10% fetal bovine serum (FBS, Pan-Biotech GmbH, Aidenbach, Bavaria, Germany) and mixture of antibiotics (0.1 mg/mL streptomycin/100 U/mL penicillin) purchased from Sigma-Aldrich Chemicals (Warsaw, Poland).

The effect of cold plasma on cell behavior was assessed using human adipose tissue-derived mesenchymal stem cells (ADSCs, ATCC-LGC standards, Teddington, UK) that were cultured in Mesenchymal Stem Cell Basal Medium with the addition of the components of Adipose-Derived Mesenchymal Stem Cell Growth Kit Low Serum (ATCC-LGC Standards, Teddington, UK) and antibiotics (penicillin/streptomycin). All cells were maintained at 37 °C, 95% of air humidity and 5% CO_2_.

#### 3.5.1. Cytotoxicity of the Biomaterials

The cytotoxicity of the biomaterials containing various concentrations of NPs was determined according to ISO 10993-5 using 24 h extracts of the scaffolds prepared as it was described earlier [49]. MC3T3-E1 cells were seeded into 96-multiwell plates in 100 µL of a complete culture medium at a concentration of 2.5 × 10^5^ cells/mL. After 24 h incubation, the culture medium was discarded and 100 µL of appropriate extracts of the biomaterials were added. Polypropylene extract was a negative control of cytotoxicity. The MC3T3-E1 cells were exposed to the extract for 24 h and 48 h and MTT colorimetric assay (Sigma-Aldrich Chemicals, Warsaw, Poland) was carried out to assess cell viability.

Cytotoxicity of the biomaterials was also determined by live/dead fluorescent staining of preosteoblasts cultured on the samples. Before the experiment, biomaterial samples (6 mm × 6 mm) were placed in the wells of a 48-multiwell plate and preincubated for 12 h in the complete culture medium. The preosteoblasts were seeded directly on the biomaterials at high concentration of 1 × 10^5^ cells/sample. After 72 h of culture, MC3T3-E1 cells on the surface of the biomaterials were stained with the use of Live/Dead Double Staining Kit (Sigma-Aldrich Chemicals, Warsaw, Poland) according to the manufacturer protocol. The stained cells were analyzed using confocal laser scanning microscope (CLSM, Olympus Fluoview equipped with FV1000, Olympus Polska Sp. z o. o., Warsaw, Poland).

#### 3.5.2. Osteoblast Growth on the Biomaterials

Ability of the biomaterials to support cell proliferation and growth was assessed by seeding MC3T3-E1 preosteoblasts directly on the biomaterials at low concentration of 3 × 10^4^ cells/sample. After 72 h of culture, the MC3T3-E1 cells were fixed as described earlier [49] and F-actin cytoskeletal filaments were stained with AlexaFluor635-conjugated phallotoxin (Invitrogen, Carlsbad, California, USA). Cell nuclei were stained using 0.5 μg/mL DAPI (Sigma-Aldrich Chemicals, Warsaw, Poland). Stained cells were observed using CLSM.

#### 3.5.3. Plasma Effect on Proliferation of Stem Cells on the Biomaterials

Human ADSCs were seeded directly on the biomaterials at extremely low concentration of 1.5 × 10^4^ cells/sample. After 24 h of culture (when the cells were well attached to the biomaterials), the culture medium was discarded and replaced with Hanks’ Balanced Salt solution (HBSS, Sigma-Aldrich Chemicals, Warsaw, Poland). Plasma treatment of the samples was performed using GlidArc reactor operated at the atmospheric pressure with the use of nitrogen as a substrate gas. The electrode tips were positioned at a 3 cm distance from the surface of the biomaterials and 7.33 dm^3^/min flow-rate of nitrogen was applied. ADSCs were exposed to nitrogen plasma for 16 s and left for 3 h in HBSS after treatment. Stem cells cultured on the biomaterials and maintained for 3 h in HBSS without plasma treatment served as control samples. Then, HBSS was discarded, fresh complete culture medium was added, and the cells were cultured for further 6 days with medium renewal on the 3rd day. On the 1st and 6th day, stem cells were fixed and stained as described in Section 3.5.2. The number of cells on the surface of the biomaterials was determined by nuclei counting using ImageJ software version 1.52a (Wayne Rasband, National Institutes of Health, Bethesda, Maryland, USA). The doubling time for the stem cells grown on the samples was calculated with the use of Doubling Time Computing software version 3.1.0.

#### 3.5.4. Plasma Effect on Osteogenic Differentiation of Stem Cells on the Biomaterials

Human ADSCs were seeded directly on the biomaterials at high concentration of 5 × 10^4^ cells/sample. After 24 h of culture, the cells were treated with cold nitrogen plasma as described in Section 3.5.3. Three hours after plasma treatment, HBSS was replaced with osteogenic medium (Osteocyte Differentiation Tool, ATCC-LGC standards, Teddington, UK) and the cells were cultured for a further 20 days with half of a medium renewal every 3-4 days. Markers typical of the osteogenic differentiation (Col I, bALP, and OC) were assessed in the cell lysates that were prepared according to the procedure described earlier [46]. The levels of the osteogenic markers were evaluated using human-specific ELISA kits (bALP ELISA Kit, FineTest, Wuhan, China; Collagen alpha-1(I) chain ELISA Kit, EIAab, Wuhan, China; OC ELISA Kit, EIAab, Wuhan, China). The total protein content was also determined for each lysate using BCA Protein Assay Kit (Thermo Fisher Scientific, Waltham, Massachusetts, USA) to normalize the amount of osteogenic markers (ng) per mg of total cellular proteins.

### 3.6. Statistical Analysis

Cell culture tests were performed in at least three independent experiments (*n* = 3). Statistical significance was considered at *p* < 0.05 and determined using One-way ANOVA followed by Tukey′s test (GraphPad Prism 8.0.0 Software, GraphPad Software Inc., California, CA, USA).

## 4. Conclusions

Within this study it was observed that extrinsic H_2_O_2_ generated by nitrogen plasma and intrinsic H_2_O_2_ generated by the presence of Fe_x_O_y_-free MSNPs in the scaffold positively affected expression of OC gene, but did not compensate the negative effect of MSNPs presence for the expression of bALP and Col I genes. The addition of Fe_x_O_y_ catalysts in MSNPs always lead to a significant increase of the quantity of reactive species (mainly short-lived species like OH radicals). The presence of these additional short-lived species had a positive effect on bALP and Col I markers but a negative effect on OC gene expression, where the concentration of reactive species was already at its optimum without the presence of the Fe_x_O_y_ catalysts.

Presented results clearly demonstrated that short-time (16 s) exposure of ADSCs to nitrogen plasma was non-toxic, accelerated proliferation of cells grown on the biomaterial containing Fe_x_O_y_/MSNPs catalyst, and increased OC production by the cells cultured on the scaffold containing MSNPs without Fe_x_O_y_ decoration. Plasma activation of the biomaterial containing Fe_x_O_y_/MSNPs catalyst resulted in the formation of sufficient amounts of active long-lived species with enhanced local generation of OH radicals, thanks to the Fe_x_O_y_ catalysts, that had positive impact on stem cell proliferation and at the same time did not negatively affect their osteogenic differentiation. Therefore, plasma-activated Fe_x_O_y_/MSNPs-loaded biomaterial is characterized by improved biocompatibility and has great clinical potential to be used in regenerative medicine applications to improve bone healing process.

## Figures and Tables

**Table 1:** Atomic composition (at. %) and atomic ratio from XPS for the Fe_x_O_y_/MCM-48 and Fe_x_O_y_/MSNPs samples, compared with pure MCM-48 and MSNPs.

	Fe_x_O_y_/MCM-48	Fe_x_O_y_/MSNPs	MCM-48	MSNPs
O 1s	64.24	68.80	68.99	60.50
Si 2p	33.41	29.18	29.74	30.87
O/Si	1.92	2.36	2.32	1.96
Fe 2p3/2	0.32	0.61	/	/
Fe/Si	0.01	0.02	/	/
C 1s	2.03	1.41	1.27	8.63

**Table 2:** Total porosity as measured by Mercury Intrusion Porosimetry together with the elastic modulus and the work of fracture (WOF) of the different materials obtained from the compression tests.

Reference	Total Porosity (%)	Young’s Modulus (GPa)	WOF (MPa.m^1/2^)
Control Biomaterial	50.1	3.92 ± 0.75	1.72 ± 0.24
MSNPs_0.25	50.4	3.68 ± 0.78	1.63 ± 0.39
Fe_x_O_y_/MSNPs_0.25	50.2	3.18 ± 0.10	1.42 ± 0.21

## Supplementary Materials

Supplementary materials can be found at https://www.mdpi.com/1422-0067/21/13/4738/s1.

## Abbreviations

ADSCs	Adipose tissue-derived mesenchymal stem cells
bALP	Bone alkaline phosphatase
Col I	Type I collagen
CTAC	CetylTrimethylAmmonium chloride
FGF-2	Fibroblast growth factor-2
HA	Hydroxyapatite
HBSS	Hanks’ Balanced Salt solution
ICP	Inductively coupled plasma
GAD	GlidArc plasma reactor
MCM-48	Mobil Composition of Matter No. 48
MIP	Mercury Intrusion Porosimetry
MSNPs	Mesoporous silica nanoparticles
NPs	Nanoparticles
OC	Osteocalcin
PBS	Phosphate buffered saline
PP	Polypropylene
RONS	Reactive oxygen and nitrogen species
ROS	Reactive oxygen species
SEM	Scanning electron microscopy
SSA	Specific surface area
TEA	TriEthanolAmine
TEM	Transmission electron microscopy
TEOS	Tetraethyl orthosilicate
XPS	X-ray photoelectron spectroscopy
XRD	X-ray diffraction
