# Supplementary material for: Positive Effect of Cold Atmospheric Nitrogen Plasma on the Behavior of Mesenchymal Stem Cells Cultured on a Bone Scaffold Containing Iron Oxide-Loaded Silica Nanoparticles Catalyst
Source: Int J Mol Sci. 2020 Jul 3;21(13):4738. doi: 10.3390/ijms21134738 (PMC7369831; doi:10.3390/ijms21134738)
Supplement: Supplementary file 1 [file ijms-21-04738-s001.pdf]

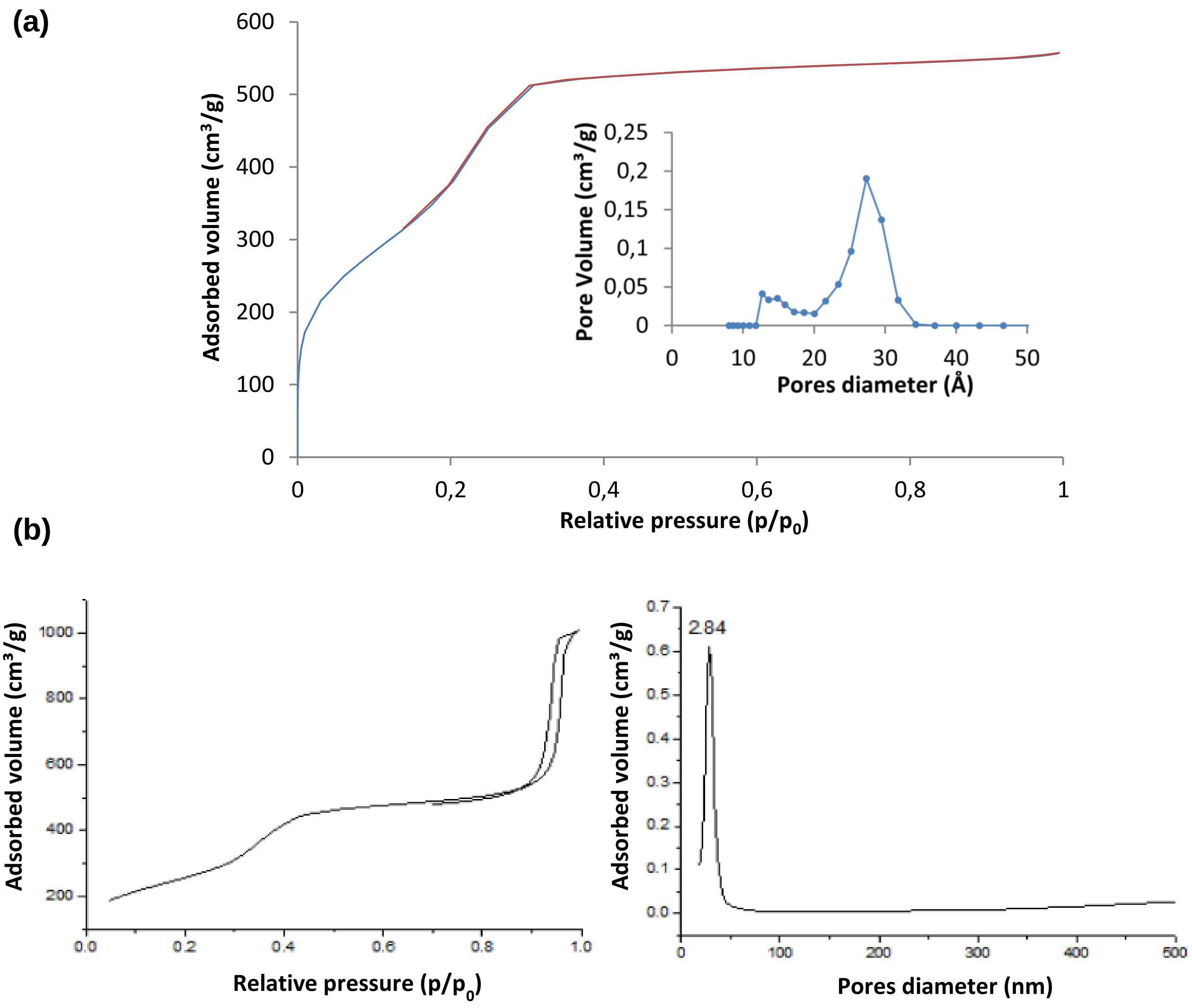

SM1. Nitrogen adsorption/desorption isotherms at 77 K of the (**a**) MCM-48 synthesized material with (inset) DFT pore size distributions obtained from N_2 physisorption curve (adsorption) and (**b**) of the MSNPs material.

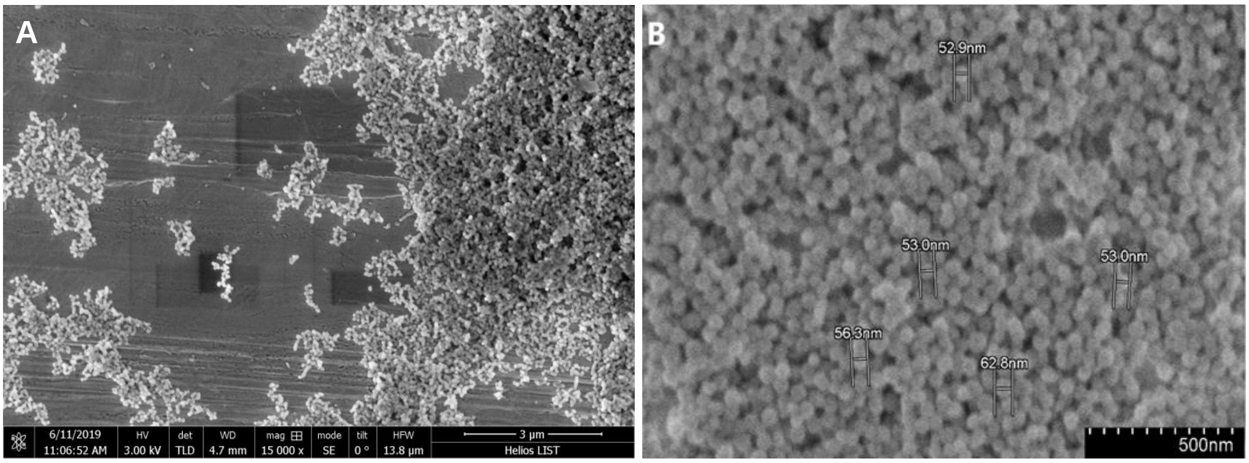

SM 2. SEM image of MSNPs showing a narrow size dispersion.

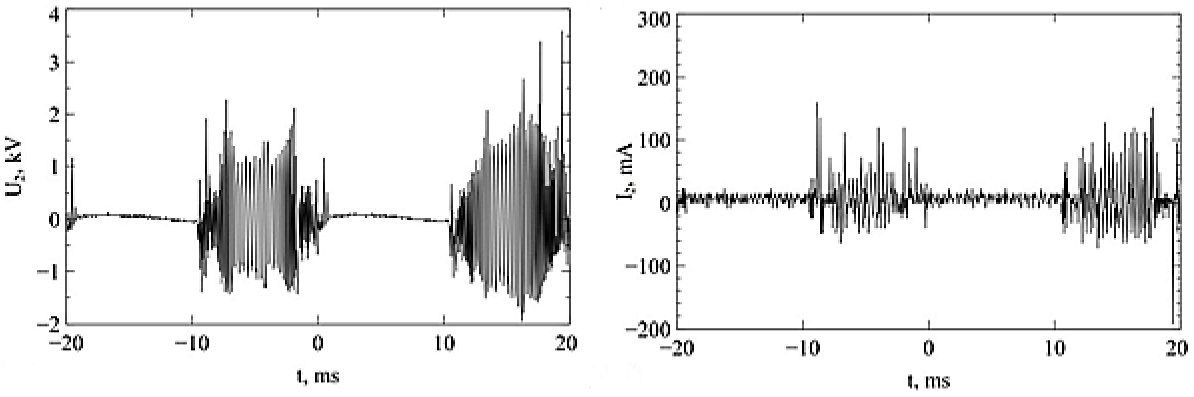

SM 3. GAD voltage and current characteristics (secondary side of transformer).
